# Differential translation elongation directs protein synthesis in response to acute glucose deprivation in yeast

**DOI:** 10.1080/15476286.2022.2065784

**Published:** 2022-05-01

**Authors:** Anna R. Guzikowski, Alex T. Harvey, Jingxiao Zhang, Shihui Zhu, Kyle Begovich, Molly H. Cohn, James E. Wilhelm, Brian M. Zid

**Affiliations:** a Division of Biological Sciences, University of California, San Diego, CA, USA; b Department of Chemistry & Biochemistry, University of California, San Diego, CA, USA

**Keywords:** Translation regulation, ribosome profiling, glucose starvation, translation elongation, ribosome runoff

## Abstract

Protein synthesis is energetically expensive and its rate is influenced by factors such as cell type and environment. Suppression of translation is a canonical response to stressful changes in the cellular environment. In particular, inhibition of the initiation step of translation has been highlighted as the key control step in stress-induced translational suppression as mechanisms that quickly suppress initiation are well-conserved. However, cells have evolved complex regulatory means to control translation apart from initiation. Here, we examine the role of the elongation step of translation in yeast subjected to acute glucose deprivation. The use of ribosome profiling and *in vivo* reporter assays demonstrated elongation rates slow progressively following glucose removal. We observed that ribosome distribution broadly shifts towards the downstream ends of transcripts after both acute and gradual glucose deprivation but not in response to other stressors. Additionally, on assessed mRNAs, a correlation existed between ribosome occupancy and protein production pre-stress but was lost after stress. These results indicate that stress-induced elongation regulation causes ribosomes to slow down and build up on a considerable proportion of the transcriptome in response to glucose withdrawal. Finally, we report ribosomes that built up along transcripts are competent to resume elongation and complete protein synthesis after readdition of glucose to starved cells. This suggests that yeast has evolved mechanisms to slow translation elongation in response to glucose starvation which do not preclude continuation of protein production from those ribosomes, thereby averting a need for new initiation events to take place to synthesize proteins.

**Abbreviations:** AUG: start codon, bp: base pair(s), CDS: coding sequence, CHX: cycloheximide, eEF2: eukaryotic elongation factor 2, LTM: lactimidomycin, nt: nucleotide, PGK1: 3-phosphoglycerate kinase, ribosomal biogenesis: ribi, RO: ribosome occupancy, RPF: ribosome protected fragment, TE: translational efficiency

## Introduction

Unicellular organisms, such as the budding yeast *Saccharomyces cerevisiae,* divide quickly when environmental conditions are favourable, including under standard laboratory conditions where yeast is cultured in glucose-rich liquid media. When glucose levels are high, robust expression of glycolytic mRNAs is well-coordinated [1–3]. This allows yeast to take advantage of favourable conditions, ferment, and divide exponentially. Rapid growth requires a massive investment of cellular energy into new protein synthesis [4]; however, organisms must respond to adverse changes in their environment and adapt gene expression programmes to survive stress [5].

An important component of the response to new environmental stress is regulation and reduction of protein synthesis from pre-existing cytoplasmic mRNAs [6]. Logically, reduced translation tends to follow acute stress because the existing transcriptome is no longer programmed for survival under current conditions. In addition, reducing translation from mRNAs encoding proteins that facilitate growth is prudent at the onset of severe stress as it circumvents the time required for nuclear changes in transcription to impact gene expression. Lowering translation also reduces energy consumption and is therefore considered a general hallmark of post-transcriptional gene regulation when dividing cells encounter stress.

Decades of research have parsed mechanisms that limit protein synthesis in response to acute stresses [7]. A great deal has focused on initiation as it is reported to be rate-limiting during growth [6, 8, 9]. Less attention has been focused on other steps of translation, although it is becoming increasingly appreciated that cells have evolved regulatory steps to modulate translation during stress that extend beyond initiation-based mechanisms. For example, eEF2, the protein that catalyzes GTP-dependent ribosome translocation during the elongation step of protein synthesis, has been shown to be phosphorylated in response to acute hyperosmotic and oxidative stresses in yeasts [10–12]. Phosphorylation reduces eEF2 activity, thereby attenuating elongation and protein production generally [13, 14]. Mammalian systems rely on eEF2 phosphorylation via eEF2 kinase to adapt to nutrient deprivation and ribosomal stress [15–17]. In response to the stress of heat shock, researchers have shown that mammalian and yeast cells globally accumulate ribosomes close to their start codons, approximately 60–100 nucleotides downstream of the translation start site. This result suggests these ribosomes successfully initiated but were slowed early in elongation, resulting in a build-up of very slow or stalled translation machinery [18, 19,20,]. Here, we employ the stress of acute glucose starvation in exponentially dividing, log phase yeast to characterize how elongation is regulated temporally in response to acute glucose removal and subsequent recovery.

Abrupt glucose deprivation is a particularly arduous stress for log phase yeast to face because glucose is their preferred carbon source and a key substrate in fermentative growth [21, 22]. Relatedly, understanding how simpler eukaryotic organisms have evolved to confront glucose starvation is relevant to understanding complex human diseases such as diabetes and cancer [23–25]. While it has been reported that there is extensive cessation of S^35^ methionine incorporation after 10 min of glucose starvation in yeast, researchers have also observed housekeeping mRNAs remain engaged in polysomes at both a relative and an absolute level [26, 27]. For example, ribosomes remain bound to the coding sequence (CDS) of the essential glycolytic gene *PGK1* after 10 and 15 min of glucose starvation [27, 28]. As glucose starvation leads to an extensive reduction in initiation [9, 21], this result strongly implies that elongation does not take place at pre-stress rates. If elongation did not slow, we would expect to see ribosomes run off the 1,251 nt *PGK1* CDS after approximately 3 min as the basal elongation rate of log phase yeast is reported to range between 3 and 10 amino acids per second [29, 30]. Importantly, this result seems at odds with a common narrative that ribosomes runoff mRNAs in response to severe stress. Runoff is highlighted as a crucial early step in a process that sequesters abundant, pro-growth, and pre-existing mRNAs into phase-separated granules such as stress granules [31–33]. This narrative is well-founded because ribosome runoff does occur as evidenced by the large collapse in the polysome repeatedly shown to take place on the timescale of minutes in glucose-starved yeast [21, 26, 28, 34]. *PGK1ʹ*s continued high ribosome occupancy (RO) observed concurrently with polysome collapse indicates ribosome runoff must be heterogeneous for both observations to take place at the same time. Therefore, a gene expression programme dependent on differential translation elongation may play a key role in regulating protein synthesis following glucose starvation and explain, at least in part, why some ribosomes run off transcripts and some remain bound to them.

Intrigued by this observation of heterogeneous ribosome behavior, we sought to better understand how yeast regulates protein synthesis and alters ribosome-mRNA interactions in the initial minutes following glucose starvation. In this article, we focused not only on general levels of engagement but also where ribosomes bind on mRNAs. We found that glucose starvation causes ribosomes to accumulate downstream on the 3’ ends of many mRNAs. This coincides with a progressively slower rate of elongation, a result we validated with *in vivo* approaches. Strikingly, this accumulation is not observed in response to other stresses. We also explored protein synthesis in log phase and glucose starvation conditions to further support our measurements of slowed elongation and observed that the extent of ribosome engagement on a transcript is not sufficient to predict differences in protein synthesis between pre- and post-stress conditions. Finally, we propose ribosomes that build up on transcripts can resume elongation following glucose readdition. Furthermore, successful protein synthesis can be observed from these re-engaged ribosomes independent of newly initiated ones.

## Results

### Glucose starvation shifts ribosome distribution along mRNAs

**Figure 1.**
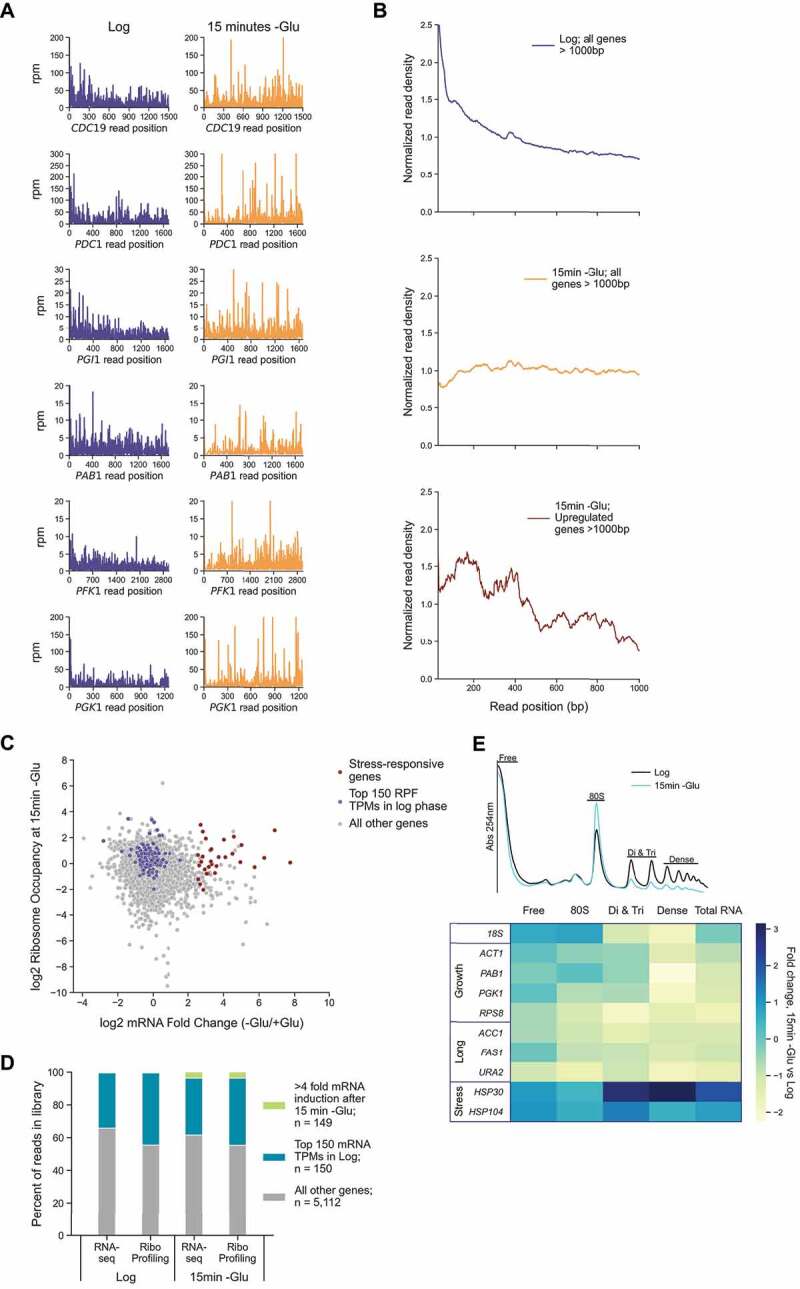
Glucose starvation alters ribosome engagement on mRNAs.

Ribosome profiling is a sequencing technique that isolates fragments of mRNAs bound by ribosomes which are turned into sequencing libraries. It is common for researchers to prepare ribosome profiling and traditional RNA-seq libraries from the same sample to calculate RO on a gene-by-gene basis and compare changes between samples and conditions. Such changes are traditionally ascribed as alterations in translational efficiency (TE) for a given gene [35–40]. However, analysis of the distribution and movements of ribosomes along transcripts at nucleotide resolution can provide deeper insight into translational regulation compared to simply considering RO changes. Using ribosome profiling, we first examined the distribution of reads, known as ribosome-protected fragments (RPFs), along mRNAs that have important roles in glycolysis and growth of a similar or longer length to the 1,251nt *PGK1* transcript (Figure 1a). Ribosomes are bound to the entire length of these mRNAs in log phase and after 15 min of glucose starvation. This indicates elongation is regulated in a way where ribosome runoff is not ubiquitous. If runoff were ubiquitous as a result of unaltered ribosome transit rate, we would expect pre-existing transcripts of this length to be largely or completely devoid of ribosomes given that new initiation and aggregate protein synthesis are markedly reduced genome-wide [9, 21].

**A**: RPF reads per million (rpm) by nucleotide position for the indicated genes during log phase (purple traces; left) and after 15 min of glucose starvation (orange traces; right). **B**: Normalized read density plots for indicated gene categories in log phase (purple line; top) and after 15 min of glucose starvation (orange line; middle and red line; bottom, respectively). To generate read density plots, the aggregate number of reads per single nucleotide position across all genes >1,000 nt with >25 reads per gene per library were included and normalized by read depth to enable inter-library comparison. **C**: Plot of log2 RO calculated as RPF reads divided by mRNA reads per gene in glucose starvation conditions against log2 mRNA induction after 15 min of glucose starvation. Abundant log phase genes (purple markers) were the 150 transcripts with the highest mean TPM scores in two replicate ribosome profiling libraries. TPM = transcripts per million. For both **B** and **C**, genes with mRNA log2 fold change > 2.5 and RO log2 fold change > 0.09 were classified as upregulated in response to glucose starvation. **D**: Percentage of all reads in the indicated libraries by category. 149 genes had >4-fold increase in mRNA reads after 15 min of glucose starvation compared to log phase (green bars). Abundant log phase mRNAs (blue bars) were the 150 mRNAs with the highest mean TPM scores in two replicate RNA-seq libraries. There is substantial overlap between the top 150 RFP TPM genes in **C** (purple markers) and the top 150 mRNA TPM genes in **D** (green bar) with 123 shared. **E**: Traces of polysome fractionation gradients showing what fractions were combined (top panel). Pooled fractions underwent RNA extraction and RT-qPCR for the indicated genes to quantify the changes in transcript abundance in each fraction after 15 min of glucose starvation and log phase (bottom panel). An exogenous, spike-in RNA was used to quantify relative abundance in each pool and the fold change in RNA level was assessed by ∆∆Ct analysis.

We were struck by the shift in the pattern of RPF reads along these genes from the upstream 5’ end in log phase towards the downstream 3’ end during stress. Plotting the distribution of read density along thousands of yeast transcripts revealed a general shift away from the start codon when compared to log phase. This indicates strong repression of translation initiation (Figure 1b; top and middle panels). This more general increase in downstream read distribution further supports the notion that ribosome runoff is heterogeneous, given that polysome collapse also occurs in glucose starvation conditions. Importantly, a group of stress-induced genes known to be upregulated transcriptionally and translationally mirror the distribution pattern observed during log phase (Figure 1b; bottom panel). These stress-responsive genes, mostly heat shock proteins, display a decreasing or negative ramp of distribution reported to be characteristic of well-translated genes [41]. These stress-induced genes evade the general halt in initiation that occurs during glucose starvation and demonstrate induction of a stress response.

We next assessed whether this small group of stress-responsive genes, which are uniquely upregulated in response to stress, have greater RO values after 15 min of glucose starvation compared to the rest of the transcriptome. If genes that are well-translated in log phase underwent massive runoff during stress, we would expect lower occupancy on those transcripts and higher occupancy on upregulated genes. Surprisingly, while transcriptional induction of stress-responsive mRNAs is very high compared to the entire genome, the magnitude of their RO at 15 min starvation did not vary from other genes, including the 150 genes that were most highly engaged with ribosomes during log phase growth (Figure 1c). We also calculated the proportion of pre-existing versus stress-induced mRNAs in our samples (Figure 1d). Stress-induced transcripts made up a relatively small proportion of libraries after 15 min of starvation. Moreover, the 150 most abundant mRNAs during log phase maintain high RPF and mRNA read counts after 15 min of stress compared to both stress-induced genes and the rest of transcriptome, further highlighting their sustained engagement with ribosomes.

Next, because ribosome profiling and RNA-seq quantify relative changes in RPF and mRNA abundances, we used polysome profiling to assess absolute changes in ribosome engagement to more rigorously test our hypothesis that high RO on pre-stress mRNAs continues during stress. Polysome profiling was performed on log phase and glucose starved samples, followed by RNA quantification of selected genes with normalization to an exogenous spike-in RNA. Fractions were pooled and five groups were analysed: a total RNA pool, a free RNA pool, a monosome pool, and two polysome fractions made of a combined disome plus trisome pool and, finally, a dense polysome pool (Figure 1f). The concentrations of pro-growth, abundant log phase mRNAs in glucose-starved polysomes were roughly twofold lower than in glucose replete, log phase polysomes. This targeted approach corroborated our ribosome profiling results. While it is evident that some transcripts do undergo runoff and leave the polysome, there is incomplete ribosome runoff during glucose starvation and many transcripts remain in heavier fractions. In addition, the polysome collapses but does not do so completely. If ribosome runoff was a straightforward, universal explanation for how translation is regulated in response to glucose starvation, we would expect the shift of abundant growth genes out of heavier polysomes to be much greater. We also quantified the presence of stress-induced mRNAs and mRNAs longer than 6,000 nucleotides in the various fractions. Considering the median yeast gene length is 1,280 nt, we considered genes more than 6,000 nt extremely long. If elongation remained near the basal rate of 3–10 amino acids per second, we would expect growth genes to shift out of heavier fractions to a greater extent than extremely long genes do because long genes cannot undergo as much runoff following a 15 min starvation timepoint. Our results indicate that long genes and growth genes leave the polysome at similar, modest rates. Our data also agree with previous polysome profiling that showed the continued presence of *PGK1* mRNAs in polysomes during acute glucose starvation [26, 28]. Together, our ribosome profiling and polysome profiling experiments highlight that ribosomes remain engaged with a population of pre-existing mRNAs during glucose starvation.

### Ribosome polarity analyses reveal that changes in ribosome distribution are stress-specific and an increase in downstream ribosome accumulation is unique to glucose stresses

**Figure 2.**
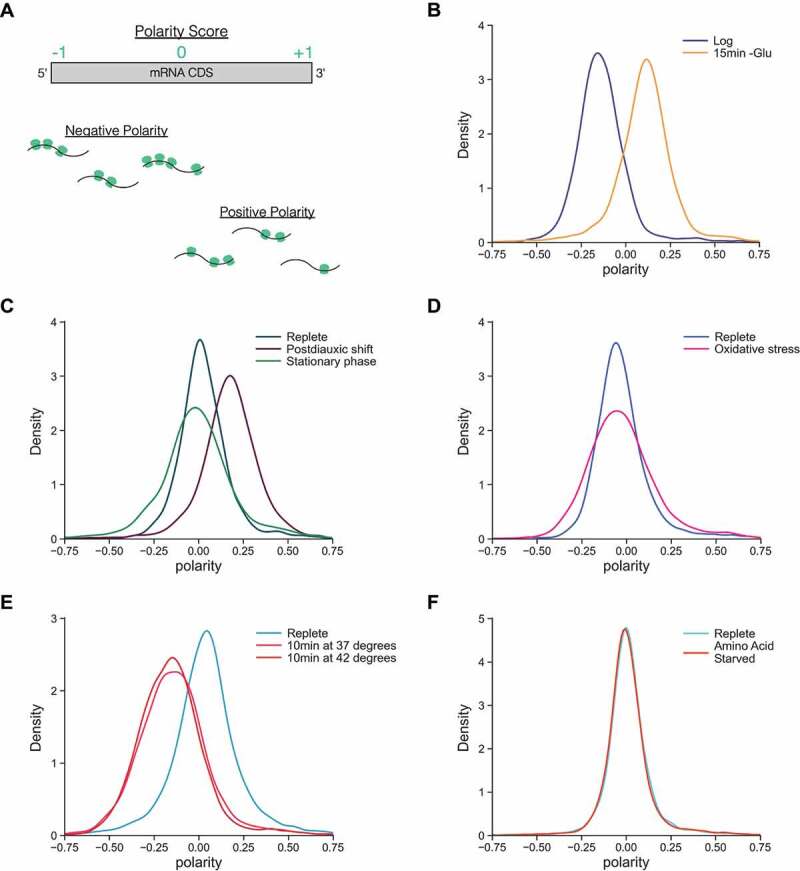
Polarity score analyses of yeast stress response ribosome profiling libraries show effects specific to glucose starvation.

Next, we sought to gain insight into genome-wide ribosome distribution along transcripts and quantify the build-up of ribosomes along mRNAs. To accomplish this, ribosome polarity scores for individual genes were calculated before and after glucose starvation [42, 43] (Figure 2a). Plotting polarity score distribution densities revealed a shift from negative to positive where more ribosomes occupy the 3’ halves of CDSs relative to 5’ halves after 15 min of starvation (Figure 2b). This suggests elongating ribosomes could slow over time and consequently remain bound to mRNAs in downstream regions during glucose starvation.

**A**: Schematic and cartoons showing how polarity scores are determined on a per gene basis. RPF reads on the 5’ half of a transcript contribute to negative polarity while reads on the 3’ half of a transcript contribute to positive polarity. **B**: Densities of polarity score distributions from log phase and 15 min acute glucose starvation. **C**: Densities of polarity score distributions from yeast culture in log phase (replete), after 1 day of growth (postdiauxic shift), and after 5 days of growth (stationary phase). **D**: Densities of polarity score distributions from pre- and post-acute oxidative stress. **E**: Densities of polarity score distributions from log phase cells before heat stress (replete) and after the indicated heat shock. **F**: Densities of polarity score distributions from pre- and post-acute amino acid starvation. For **B-F,** per-gene polarity scores were calculated from genes that had >25 reads per library and plots were generated from the distribution of these scores. Although not at identical densities, all replete samples were in log phase as assessed by OD_600_ measurements in the range of 0.4–1.0.

Our initial ribosome profiling libraries were prepared with cycloheximide (CHX) pretreatment. Pretreatment is a technique that many labs have moved away from as it is reported to complicate the interpretation of ribosome distributions around the start codon and bias TE measurements on yeast transcripts, particularly ribosomal biogenesis (ribi) mRNAs [44, 45]. At the same time, foregoing CHX pretreatment causes artifactual ribosome runoff near the AUG in log phase samples because harvesting cells out of nutrient-rich media is inherently stressful [36] (Figure S1). We hoped to explore how substantially including or excluding pretreatment impacted nucleotide-resolution RO in the context of glucose starvation and to expand our analysis with an approach that would enable us to interrogate the dynamics of ribosome movement. To accomplish this, we prepared replicate libraries without CHX pretreatment at log phase, 1, 5, 10, 15, 20, and 30 min time points, as well as a matched 15-min glucose starvation sample that underwent 1 min of pretreatment before harvest as was performed on our earlier libraries. It was previously reported that, following 20 min of amino acid starvation, a 2 min CHX pretreatment followed by a 3 min centrifugation and resuspension into lysis buffer caused a CHX-dependent decrease in footprints on ribi mRNAs when compared to harvest via vacuum filtration without pretreatment [45]. Contrastingly, when we compared our 15-min glucose starvation timepoint samples with or without pretreatment, we see no difference in the global footprint counts with an R^2^ > 0.99 (Figure S2). This includes no significant differences in ribosome loading on the ribi gene class. Furthermore, the distribution of ribosome density along mRNAs and polarity score distributions have negligible differences between the pretreated and untreated 15-min timepoints (Figure S1). These results suggest the impact of pretreatment during ribosome profiling experiments varies based on the stress conditions used.

Because we saw minimal differences in ribosome loading and distribution during glucose starvation between our matched pretreatment and untreated samples, we moved forward with a polarity analysis of this time course. This showed that polarity shifts positive within 1 min of glucose starvation even without CHX pretreatment, but the magnitude of this shift does not continue to increase over time proportional to the amount of time elapsed (Figure S3). This result added nuance to our earlier hypothesis that ribosome elongation slows during glucose starvation by suggesting that it does so increasingly with time. Ribosomes appear to move quickly at the onset of starvation, rapidly shifting polarity positive, but do not continue doing so uniformly as stress induction increases from one to several minutes. This, in turn, suggests that the regulation of elongation is altered and slowed over time. This would enable runoff in the initial seconds following stress, particularly on short genes which inherently have less sequence space for ribosomes to move along before translation terminates. Our findings also shed light on how, simultaneously, longer transcripts such as those in Figure 1 remain occupied by downstream ribosomes after 15 min of starvation.

To further parse and confirm that ribosome transit slows progressively, the distribution of reads in the time course was plotted and we compared the magnitude in shift of read density between different starvation timepoints (Figure S4). Notably, we observed a striking difference in ribosome engagement around the start codon between libraries prepared either with or without CHX pretreatment in log phase. We also used the temporal nature of our time course to quantitatively measure how ribosome transit rates change between different time points (Figure S5). Finally, we calculated how polarity score on a per gene basis changes over time during starvation as a function of gene length (Figure S6). Collectively these analyses indicate that, as glucose starvation progresses, the average time needed for ribosomes to move along mRNAs increases. Both the magnitude of the drop in read density that occurs near the start codon and the magnitude of how polarity scores change between samples are not proportional to time elapsed between sample collection. These additional analyses lend support to our initial observation that ribosomes build up along transcripts due to a decrease in transit during glucose starvation. Taken together, they point towards a regulated system whereby ribosome movement slows temporally and, globally, ribosomes move more slowly as starvation time increases. Additionally, the consequences of foregoing pretreatment while harvesting and flash-freezing log phase yeast samples for library preparation are reflected near the start codon in read distribution plots. These show a drop in read density which would not be expected without stress induction caused by media removal and therefore reveal unintended biases that can be introduced by skipping pretreatment.

Next, we were curious if this build-up of ribosomes downstream on mRNAs, which we hypothesize reflects progressively decreasing ribosome transit, was a general response to stress. We prepared ribosome profiling libraries and plotted polarity in response to multi-day growth in cells as they transitioned from log phase to postdiauxic shift to stationary phase (Figure 2c). Additionally, we performed a polarity score analysis on published profiling libraries of oxidative stress, heat shock, and amino acid starvation samples (Figure 2d–f) [12, 18, 45]. Strikingly, we found that the distribution of polarity scores did not change for amino acid starvation and oxidative stresses while heat shock showed a negative shift in polarity, corroborating reports that ribosomes build up close to the start codon during this stress [18, 19]. The only positive change in polarity we observed in addition to acute glucose starvation was from day-old yeast cultures grown to postdiauxic shift conditions. Moreover, polarity shifted back to a distribution near 0 in our sample prepared after 5 days of growth when cells were in stationary phase. This indicates that, for the stresses analysed, ribosomes uniquely build up on the 3’ half of transcripts in conditions in which glucose is newly limited, either via acute removal from the media or upon a switch to ethanol utilization that results from consumption of glucose over time causing the diauxic shift.

### In vivo *measurements show that elongation slows and the relationship between ribosome engagement and protein production changes after glucose starvation*

**Figure 3.**
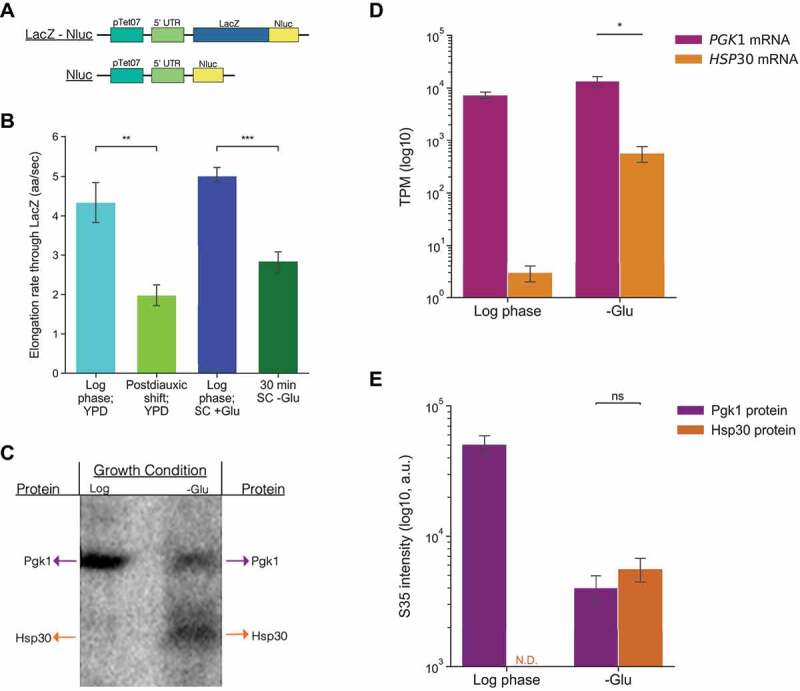
Glucose starvation impacts protein production in living cells by slowing elongation and altering the relationship between ribosome engagement and translation.

We wanted to further test the hypothesis that, in response to glucose-limited conditions, ribosomes slow down and build up on the downstream ends of CDSs because of decreased elongation by directly measuring elongation rate. To accomplish this in living cells, we developed an inducible reporter assay that enabled us to calculate the time needed for elongation through a region upstream of a yeast-optimized Nanoluciferase (Nluc) reporter gene [46]. The assay is designed to allow for comparison between the time needed to detect luciferase signal from a Nluc-only reporter to a second reporter, LacZ-Nluc, that is identical except it has an exogenous, long open reading frame (LacZ; 3,072nt) fused in front of the luciferase CDS (Figure 3a). An analysis technique known as Schleif plotting, which factors both reporter induction and the amount of time that elapses between expression of the Nluc and LacZ-Nluc reporters, was used to calculate the average elongation rate necessary to translate through LacZ [47, 48]. Utilizing this assay, we calculated elongation rate to be significantly decreased in cells subjected to acute glucose deprivation and postdiauxic shift conditions, respectively, relative to log phase (Figure 5b). Additionally, the elongation rates we found in log phase were consistent with previously reported rates [29, 30].

**A**: Schematic of reporters used to determine elongation rates in **B**. **B**: The elongation rate in amino acids per second (aa/s) through LacZ calculated following reporter induction in the indicated growth and media conditions. **C**: Representative image of autoradiography exposure used to calculate radiolabeled S^35^ methionine incorporation in **E**. For each lane, immunoprecipitation was performed on the indicated TAP-tagged proteins from cell lysates grown to log phase and, for the right lane, glucose starved for 30 min. The volume of lysate loaded for the log phase sample (left lane) was 1/10^th^ the volume loaded of the starved lysate. **D**: The TPM of RPF reads from replicate log phase and glucose starvation ribosome profiling libraries plotted as mean ± SEM. **E**: S^35^ intensity from four biological replicates performed as described in **C**. a.u. = arbitrary units. In **B**, **C,** and **E** values are plotted as mean ± SEM from a minimum of four biological replicates. Statistical significance was assessed by unpaired Student’s t-test (***p < 0.001; **p < 0.01, * p < 0.05 and ns = not significant).

We also directly examined the relationship between RO and protein production before and after glucose starvation. In general, it is often assumed that RO calculations from profiling data correlate with protein production in such a way that genes with high RPF read counts and ROs have high levels of protein synthesis, hence the term ‘translational efficiency’. We were curious if the ribosomes that occupy an abundant pre-stress gene such as *PGK1* produce less protein than those occupying an upregulated, stress-responsive gene such as *HSP30*. To test this, we added TAP tags to both and performed immunoprecipitations from log phase and glucose starvation cultures supplemented with S^35^ methionine (Figure 3c). Quantification of protein production revealed that, during log phase when *HSP30* has very few ribosome counts, we were unable to detect protein production above background while ribosomes bound to *PGK1* showed robust protein production (Figures 3d-e). Therefore, a consistent relationship exists between RPF reads and protein production in the absence of stress. However, during glucose starvation, despite *PGK1* having about 25-fold higher RPF counts along its transcripts, there was not a significant difference in S^35^ incorporation into Pgk1 and Hsp30 proteins. Together, this indicates that differential elongation during glucose starvation results in divergent levels of protein production in a gene-dependent manner. Importantly, this highlights that careful consideration must be made prior to assuming high levels of ribosome-mRNA interactions on a given transcript necessitate robust translation of that mRNA and that caution that RO is not always synonymous with TE.

### Glucose readdition causes translation to increase and elongation to proceed from ribosomes that built up on long mRNAs during glucose starvation

Finally, we sought to establish whether the ribosomes that slow or stall along mRNAs during glucose starvation are competent to resume translation after glucose is added back to the environment. To do this, we utilized both ribosome profiling and *in vivo* reporter assays. After acute glucose starvation, glucose was added to the media, and the samples were collected for ribosome profiling, RNA-seq, and polysome profiling after 1 and 5 min (Figure S7). We were particularly interested in extremely long genes which we expected to be poorly translated during glucose starvation but were earlier shown to remain associated with the polysome as assessed by qPCR (Figure 1e). Indeed, the change in RO for all but two genes greater than 4,000 nt is above 1 during glucose starvation compared to log phase (Figure S8). In general, longer genes have higher relative occupancy during starvation compared to shorter genes. This observation is consistent with our finding that ribosome elongation slows progressively in response to glucose starvation which means shorter genes are more likely to undergo runoff and have a decrease in occupancy.

**Figure 4.**
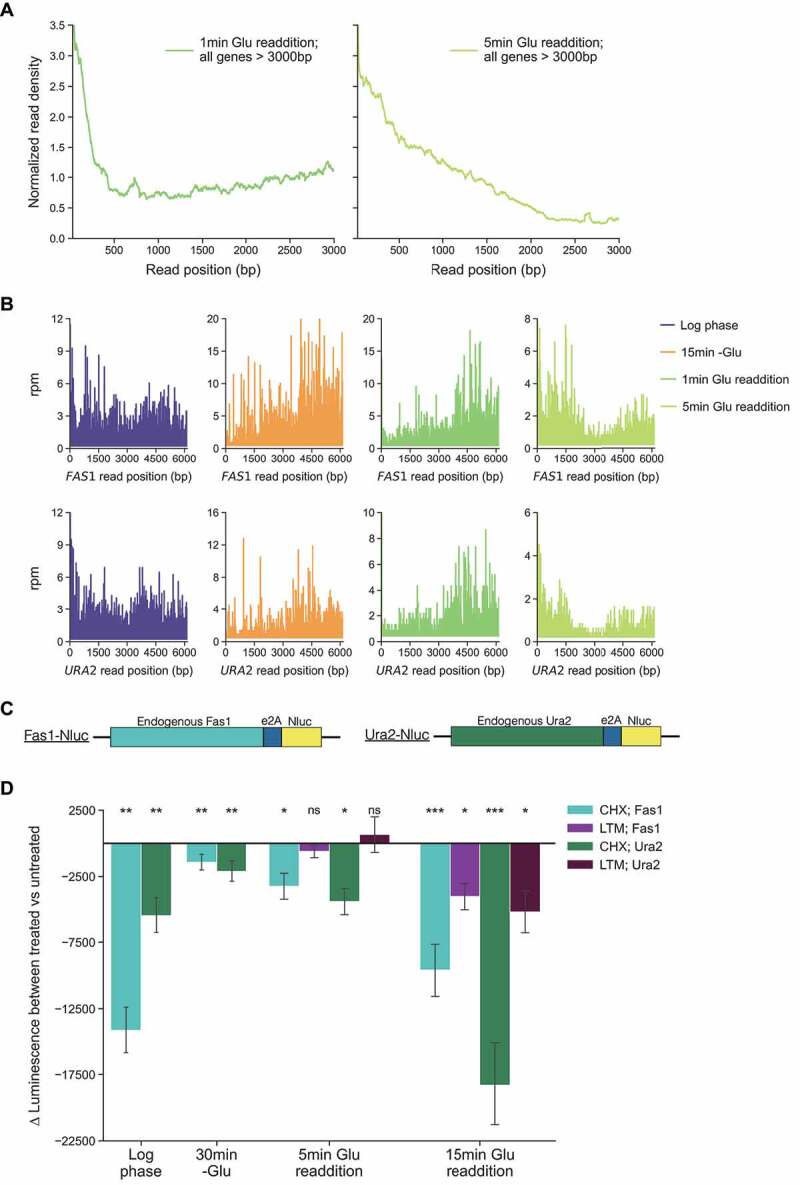
Glucose readdition following starvation results in new initiation and continued elongation.

Upon glucose readdition, a ‘wave’ of increased RPF density, suggestive of new initiation events, was detected near the start codons of long genes within the first minute (Figure 4a). By 5 min, this wave of newly initiated ribosomes was observed spanning the first approximately 2,200 nt of mRNAs. To assess ribosome movement in response to glucose readdition in a gene-specific manner, we looked at the distribution of reads on two yeast genes that are particularly long, each over 6,000 nt, in log phase, starvation, and readdition conditions (Figure 4b). We wondered whether it would be possible to parse the movement of ribosomes that slowed on these mRNAs during starvation from those that were newly initiated. Intriguingly, the distribution of ribosomes bound during glucose starvation appears to move down the transcript at the same time as new initiation events occur, resulting in a bimodal distribution of RPF reads at the 5’ and 3’ ends of these genes after 5 min of readdition. We hypothesized that there could be two populations of ribosomes on the CDSs: one population of ribosomes that underwent initiation during log phase, built up downstream during glucose starvation, and then resumed elongation and a second population that were newly initiated following glucose readdition. This led us to wonder if we could directly test whether the former were actively elongating ribosomes and, furthermore, whether these ribosomes could finish translation and produce functional protein.

**A**: Normalized read density plots for glucose starved cultures after 1 min (green line; left) and 5 min (yellow line; right) of glucose readdition. To generate read density plots, the aggregate number of reads per nucleotide position across all genes >3000 nt with >25 reads per gene were included and normalized to enable inter-library comparison. **B**: RPF reads per million by position for the indicated genes in log phase (purple trace), glucose starvation (orange trace), after 1 min readdition (green trace) and 5 min readdition (yellow trace). **C**: Schematic of reporters used to determine luciferase production in **D. D**: Each bar represents the mean difference ± SEM in luciferase signal detected during measurement between aliquots of untreated culture and the same culture treated with the indicated translation inhibitor from a minimum of six biological replicates. For log phase and 30 min-Glu conditions, the signal was recorded after 5 min of treatment and the difference was plotted on the y-axis. For readdition, the signal difference was taken at the indicated time points following glucose addition. Statistical significance was assessed by paired t-tests for differences in luciferase production between cultures that underwent either LTM or CHX treatment, respectively, paired against luciferase production from the same culture without treatment (***p < 0.001; **p < 0.01, * p < 0.05, ns = not significant).

To test the potential for these ribosomes to resume translation upon relief of starvation, we tagged the long, endogenous *FAS1* and *URA2* genes with an E2A self-cleaving peptide followed by NLuc and monitored reporter expression in log phase, during glucose starvation, and following glucose readdition [49] (Figure 4c). We used a Nluc reporter fused to a PEST domain. This allowed a short-lived luciferase reporter, with a reported half-life of approximately 5 min, to monitor recent protein production without perturbing the function of the endogenous, upstream Ura2 or Fas1 proteins following their cleavage via E2A [46]. We estimated these mRNAs were long enough that any new translation events would take longer than 5 min to complete as the ‘wave’ of ribosome density we saw in Figure 4a would correspond to translation of proteins less than 1,000 amino acids. Additionally, once initiated, a ribosome would need to elongate at 7–8 amino acids per second to translate through these reporters within 5 min. This rate is faster than the elongation rates we observe even in log phase conditions (Figure 3b) and faster than the rate we would predict from our ribosome profiling data. Even still, to separate translation events that arise due to new initiation after glucose readdition from translation events by ribosomes that completed initiation prior to readdition, we developed an experimental approach that directly decoupled these two possibilities.

Specifically, we glucose starved cells expressing these reporters for 30 min, added glucose back, and then measured luciferase production in the presence (treated) and absence (untreated) of two different drugs: either CHX, a translation elongation inhibitor, or lactimidomycin (LTM), a translation inhibitor that preferentially inhibits the initiation step of translation at the concentration used [50, 51] (Figure 4d). Since CHX addition prevents ribosomes from completing elongation and producing any functional luciferase, the difference in luciferase signal between the CHX-treated versus untreated samples represents all luciferase produced during signal measurement. In all conditions tested, there was a significant difference in luciferase signal with CHX treatment compared to untreated cultures, indicating expression was taking place in all conditions. As expected, expression was greatly reduced in starvation conditions compared to log phase for both reporters. Intriguingly, after 5 min of glucose readdition, there was no difference in protein expression due to LTM treatment compared to the untreated samples. This suggests the Nluc expression that took place did not depend on new initiation events. If it had, using an initiation inhibitor would have reduced luciferase production compared to the untreated sample. On the other hand, we found that upon 15 min of glucose readdition, significantly less protein was produced from both CHX and LTM treatments. This suggests new initiation events were contributing to expression after 15 min, unlike after 5 min. Taken together, we interpret these results to demonstrate that there is indeed a population of ribosomes bound to the CDSs of our reporters that underwent initiation prior to glucose readdition, slowed or stalled during stress, resumed elongation upon glucose readdition, and ultimately produced functional protein.

## Discussion

Here, we explored the distribution of ribosomes across yeast mRNAs during acute glucose starvation to better understand how yeast regulates protein synthesis during stress. Notably, we found that many pro-growth mRNAs retain relatively robust RO, but the distribution of these ribosomes skew 3’ and they have positive polarity. These results contrast with those for well-translated stress-induced genes like *HSP30*. We hypothesize that this altered ribosome distribution is driven by cessation of initiation followed by progressive slowing of elongation. Examining this observation concordantly with reports of polysome collapse during glucose starvation leads us to posit a nuanced interpretation of how ribosomes run off in response to glucose starvation. Specifically, in the initial seconds following glucose removal, elongation continues at a rate comparable to pre-stress, log phase elongation. This rapid ribosome movement causes ribosomes to finish translating shorter genes, which we show are more likely to display a decrease in RO, as their short CDSs inherently require less time for runoff to take place. Then, as the duration of acute stress continues and seconds turn to minutes, ribosome transit slows more and more. This leads to an accumulation of downstream ribosome binding on mRNAs of sufficient length such as *PGK1*. Meanwhile, shorter genes are more devoid of ribosomes. As the shortest yeast mRNAs tend to code for ribosomal proteins and ribosomal biogenesis genes, we conclude one way yeast responds to acute glucose starvation and downregulates bulk protein synthesis quickly is through reducing expression of these short transcripts and halting production of new translation machinery. Conversely, comparatively longer, glycolytic genes like *PGK1* remain in the polysome to a larger degree, perhaps to retain the ability to more quickly produce protein if glucose is reintroduced to the environment.

Polarity score analyses of ribosome profiling libraries proved to be an effective approach to compare ribosome engagement across various stresses. A polarity analysis of a postdiauxic shift sample displayed positive polarity like that observed during glucose starvation. Notably, a positive shift in polarity was unique to glucose-limited conditions and no polarity shift was observed in response to amino acid starvation or oxidative stress. The other stress that presented an altered polarity was heat shock. It has previously been reported that widespread elongation pausing takes place towards the 5’ end of most mRNAs during severe heat shock in mammalian cells [19]. While elongation rates were not measured during heat shock, this may be the cause of the more negative polarity we noticed in yeast and indicate that elongation is regulated more quickly in response to heat shock compared to glucose starvation. Similarly, it could instead or concurrently represent a less severe reduction in initiation. Altogether, ribosome polarity score distributions are a useful proxy to explore ribosome movement before and after stress and provide additional insight not provided by RO measurements alone.

We also note the impact of foregoing cycloheximide pretreatment during ribosome profiling library preparation. For our glucose starved samples, pretreatment did not impact gene-specific RPF counts, ribosome distribution, or ribosome polarity when compared to samples that had no pretreatment. This result highlights that the elongation rate is markedly slowed at 15 min of starvation. Inhibiting translation with 1 min of CHX pretreatment had little bearing on the library because translation was already inhibited. On the other hand, RPF read density near the start codon in log phase samples is largely decreased when pretreatment is skipped compared to samples that received 1 min of pretreatment. We speculate this absence of read density in samples that were not exposed to CHX until lysis is an artifactual result from a brief stress response that resulted during the time required for vacuum filtration and cell scraping to take place. In short, unintended stress occurs between the time it takes to remove yeast from their incubator and flash freeze them. This causes runoff without CHX present to halt elongation. Effectively, our log phase cells underwent a brief period of glucose starvation during harvest as their glucose-rich media was removed. This highlights how technical nuances in each step of a protocol can impact the results of high-resolution approaches like ribosome profiling. Things that might seem trivial such as the distance between an incubator and a filtration apparatus or the strength of the vacuum line attached to that apparatus impact the amount of time it takes to harvest cells, thereby influencing how much stress is introduced prior to library preparation. Notably, other groups have reported on the complexities of interpreting CHX-induced alterations in read density [52]. In fact, the original article that describes ribosome profiling and established pretreatment also includes a comparison between pretreatment, no pretreatment and the effects of flash freezing on read density [36]. It is clear that the duration of pretreatment and the time it takes to harvest cells are critical in ribosome profiling experiments. Traditionally, CHX-pretreatment in yeast experiments was done by adding CHX to a culture followed by shaking and incubation for 2 min prior to harvest. Moreover, harvest was often done by centrifugation which typically takes longer than vacuum filtration. We posit that there might be a middle ground between this approach and foregoing pretreatment entirely, as has become commonplace. It is possible that a balance could be struck to minimize artifacts from pretreatment without inducing new artifacts from premature stress and runoff during harvest; we would recommend researchers consider adding CHX to their culture right as they begin vacuum filtration but skip the additional two minutes of pretreatment incubation. Future experiments using this approach in concert with traditional pretreatment and no pretreatment in matched cultures could provide insight into its impact on read density near the start codon and would test whether a brief CHX exposure during filtration is sufficient to prevent the runoff we observed. Such an approach might be particularly useful for researchers hoping to compare stress conditions to bona fide non-stressed controls.

In addition to ribosome profiling, we explored how glucose starvation impacts protein production in living cells. *In vivo* measurements of elongation rate using Nluc reporters showed elongation is slower during acute glucose starvation compared to log phase. Similar effects took place during the postdiauxic shift, a less acute manner of glucose starvation. While there has been a growing appreciation in recent years for the importance of translation regulation at the step of elongation, much of this work has focused predominantly on codon-specific effects as it has been well-established that certain motifs cause ribosome elongation to stall and activate ribosome quality control pathways [53–55]. Our findings are indicative of a more general phenomenon as elongation rates differ on the same reporter in a condition-dependent manner (Figure 3b). Ribosome elongation slows significantly during glucose starvation when compared to log phase growth. Our results also indicate that slowed and paused ribosomes are primed to resume elongation and finish translation if environmental conditions continue to fluctuate, but in a favorable way. Specifically, we show that long mRNAs can undergo translation from ribosomes bound before glucose readdition. Greater protein production was measured upon glucose readdition than would otherwise be expected from new initiation alone because samples treated with the initiation inhibitor LTM show no significant difference in protein expression during the first 5 min of glucose readdition. This indicates the expression we detected comes from pre-existing ribosomes. We speculate that such pausing may allow for a population of mRNAs to remain bound to ribosomes for rapid continuation of growth once stress has been relieved, provided the duration of the stress is not too long. Additionally, the shift towards positive polarity which takes place following the postdiauxic shift and acute glucose withdrawal suggests the general slowdown in elongation we identify during glucose starvation may play an important role in fine-tuning translation during metabolic transitions to alternative carbon sources and metabolic pathways more generally, though this remains to be directly tested.

It is important to note that, though we compared elongation rates along identical mRNA sequences, we did not simultaneously test how fast elongation takes place on populations of mRNAs transcribed prior to glucose starvation compared to mRNAs transcribed during glucose starvation. This is due to experimental limitations imposed by inducible reporters, an approach necessary to ensure that elongation rate calculations were not muddled by detecting protein expression from pre-stress transcription events. Given the necessity of inducing reporter expression after stress to measure elongation rates during stress, we conjecture that our calculated elongation rate, though slower than it is during log phase, still overestimates the elongation rate that would be observed for ribosomes moving along mRNAs that were transcribed pre-stress. This is based on our observation that ribosome engagement with pre-existing mRNAs remains abundant, even during glucose starvation, though protein synthesis from them is greatly reduced (Figures 3d-e). Consequently, we speculate the elongation rate of approximately two amino acids per second calculated for LacZ in glucose starvation is more comparable to the elongation rate along a stress-responsive gene such as *HSP30* which is both transcribed and translated in response to stress and is not pre-existing. The elongation rate on *PGK1*, a pre-existing transcript poorly translated during stress but with high RO, would be even slower. Future experimental approaches to parse the difference in elongation rate during stress on mRNAs transcribed pre-stress compared to mRNAs transcribed during stress would provide more insight into this nuance. In addition, future experiments that parse the molecular mechanisms yeast employ to slow elongation in response to glucose starvation are needed to determine how cells carry out this slowdown progressively over time. Overall, this work demonstrates how ribosome profiling and reporter assays can complement one another and it highlights the importance of examining read distribution instead of simply using RO counts as a proxy for TE, especially during fluctuating environmental conditions.

## Materials & methods

### Yeast strain information

Yeast strains used are listed in Supplementary Table 1. All ribosome profiling libraries including those from log phase, glucose starvation, glucose readdition, postdiauxic shift and stationary phase samples prepared for this study were made with strain BY4741 (MATa his3∆1 leu2∆0 met15∆0 ura3∆0). Strains with either TAP-tagged Hsp30 or Pgk1 were from the yeast-TAP-tagged ORF library collection [56]. For luciferase measurements during glucose readdition, the E2A-NlucPEST sequence was inserted into a pKT vector containing a hygromycin selection marker [57]. Endogenous genes were tagged with E2A-NlucPEST through the integration of PCR products including 40 nt overhangs homologous to the sequence immediately upstream and downstream of the 3’-end of the target gene. P_TetO7_-LacZ-NlucPEST and P_TetO7_-NlucPEST were assembled into a pRS305 integration vector with homology for the LEU2 locus. Polysome profiling was performed with strain ZY185. Plasmid pST1760 [58] was integrated in strain EY0690. Endogenous Dhh1 was C-terminally tagged by PCR amplification of a 3x mini auxin-inducible degron from plasmid pST1932 [58] with homology for the 3’-end of Dhh1. Yeast transformations were performed using standard growth and transformation techniques utilizing lithium acetate and PEG as previously described [59].

### Yeast growth and glucose starvation for RNA-seq and ribosome profiling

Ribosome profiling experiments were performed with strain BY4741 grown in batch culture at 30°C with shaking at 250 rpm to OD_600_ between 0.4 and 0.6 for all log phase samples. Synthetic complete (SC) media with 2% (w/v) glucose was used to grow cells for all acute starvation experiments. Glucose starvation was performed in SC media prepared without glucose (SC-G). For each starvation sample, a portion of the volume of a culture was filtered for transfer to SC-G media while the other portion remained incubating in glucose replete media in log phase, non-stressed conditions. Cells were collected with a vacuum filtration apparatus onto cellulose filter membranes and scraped with laboratory spatulas. For glucose starvation, the cells were collected, quickly rinsed in 50–100 mL of pre-warmed SC -G media, re-filtered and resuspended in pre-warmed SC -G with continued rotation at 30°C for either 1, 5, 10, 15, 20, or 30 min, as indicated. The remaining log phase cells still in SC media were harvested while starvation samples were incubating in SC-G. For glucose readdition experiments, cultures that underwent starvation were supplemented with a 2% (w/v) final concentration of glucose that was added back to the media with continued shaking at 30°C for the indicated times prior to harvest. For the multi-day growth experiment, yeast was grown in liquid YPD (2% peptone, 1% yeast extract, and 2% dextrose) instead of SC media. These samples were collected at log phase (0 day), postdiauxic shift (1 day) and stationary phase (5 days) conditions as described in Ref. [60]. Following vacuum filtration, all cells were flash frozen in liquid nitrogen and stored at −80°C until library preparation.

## RNA-seq and ribosome profiling library preparation

For the log phase, glucose readdition and glucose starvation samples that underwent pretreatment with cycloheximide, libraries were prepared as described in Ref. [27]. Briefly, prior to harvesting, CHX was added to a final concentration of 100 µg/mL for 1 min with continued shaking at 30°C. Cells were pulverized under cryogenic conditions, extracts were digested with RNase I, and RPFs were isolated from monosome fractions via sucrose gradient sedimentation. Then, 28mer RPFs were selected, polyadenylated and reverse transcribed. RNA-seq libraries from these samples were prepared following or poly(A)+-selected RNA using Oligo(dT) Dynabeads (Invitrogen), as described in 27.

Libraries that did not undergo CHX-pretreatment, including log phase, acute glucose starvation, postdiauxic shift and stationary phase samples, were prepared following methods published by Ref. [61]. Minor modifications to monosome isolation were made and are described below. Briefly, after cells were flash frozen, they were ground with yeast footprint lysis buffer (20 mM Tris-Cl [pH 8.0], 140 mM KCl, 1.5 mM MgCl_2_, 1% Triton X-100) frozen in liquid nitrogen dropwise via cryogenic ball milling in a planetary ball mill with 2 min of boiling in liquid nitrogen between cycles. Lysates were thawed, RNA was quantified, 30 µg total RNA was digested with RNase I (Epicentre), and monosomes were isolated with size exclusion chromatography [62]. RPFs were separated and size-selected via denaturing TBE-Urea PAGE. Next, footprints underwent dephosphorylation with T4 PNK and linker ligation with T4 enzyme Rnl2(tr) K227Q (NEB). Ligation reactions were excised following separation and size selection on a TBE-Urea gel and pooled. Next, pools underwent reverse transcription with Protoscript II (NEB), circularization with CircLigase II [Lucigen], quantification with qPCR and PCR amplification, as described by Ingolia & McGlincy. Libraries were sequenced at the Institute for Genomic Medicine sequencing core at UC San Diego on an Illumina HiSeq 4000.

## Ribosome profiling bioinformatic analysis

For libraries prepared with CHX-pretreatment, read trimming and alignment took place as described in Ref. [27]. For libraries prepared without CHX-pretreatment, read trimming and alignment took place as follows. First, unprocessed fastq files were trimmed with Cutadapt [63] to remove the adapter sequence with the parameter -a AGATCGGAAGAGCAC. Reads less than 17nt or without adapters were discarded. For files that required manual demultiplexing, Cutadapt was used again to demultiplex with a custom fasta containing the barcode sequence corresponding to a given biological sample. Next, Cutadapt output files had their unique molecular identifiers (UMIs) removed from the read line of the fastq and appended to the header line with a custom python script for subsequent deduplication of PCR artifacts. Next, reads were aligned to a reference of *S. cerevisiae* noncoding RNA using bowtie [64] with the following parameters: -k 1 – best -t -S -q. Reads that did not align to ncRNA were retain for alignment to the genome. First, these unaligned reads were filtered to remove low quality reads based on Phred score with fastqx_toolkit (http://hannonlab.cshl.edu/fastx_toolkit/). Those that passed this quality control step were aligned against the *S. cerevisiae* genome. Index files generated via bowtie were from genome assembly R64-1-1 (Saccharomyces Genome Database (SGD)). Next, files were deduplicated to remove PCR duplicates with custom python scripts. Read features were counted using default parameters in htseq-count [65] against gtf feature files obtained from SGD using genome assembly R64-1-1. To calculate polarity scores per gene custom python scripts were run based on methods described in 43. All scripts used to process and analyse data and generate plots are available upon request and sequencing data has been deposited at the NCBI GEO database with accession number GSE200491.

## Polysome profiling

800 mL cultures of strain ZY185 were inoculated in SC media and grown overnight to mid log phase (0.4–0.6 OD_600_). 400 mL of culture was rapidly filtered, washed, and resuspended in SC-G media to begin glucose starvation. The remaining half of the glucose replete culture was rapidly filtered, and the cell paste was scraped into liquid nitrogen for flash freezing. 1.2 mL of polysome gradient lysis buffer (20 mM Tris-Cl (pH 7.5), 140 mM KCl, 2 mM MgCl_2_, 100 µg/mL CHX, 20 U/mL SUPERase•In™ (Invitrogen), 1% Triton X-100) was flash frozen dropwise with the cell paste. After 15 min of glucose starvation, SC-G cultures were filtered down and the cell paste was flash frozen with 1.2 mL of lysis buffer. Cell pastes were stored at −80°C. Cell lysis was performed by cryogenic ball milling for 4 × 3 min cycles and cooled with liquid nitrogen between each cycle. The resulting lysates were gently thawed to room temperature in a water bath and treated with DNase I (12.5 U/mL). Lysates were centrifuged at 4°C for 5 min at 3000xg, and the supernatant was centrifuged once more for 10 min at 20,000xg. Approximate concentrations were estimated by A_260_ measurements on a Nanodrop 2000c (Thermo Fisher).

A 7%–47% sucrose gradient in polysome gradient buffer without Triton X-100 was prepared with a gradient maker. Clarified supernatants were added and centrifuged at 4°C for 3 h at 35,000 RPM in a Beckman SW41Ti rotor. The gradient was fractionated into 1 mL aliquots using a gradient fractionator and UA-6 detector (Isco/Brandel). Polysome traces were monitored through absorbance measurements at 254 nm. 2 ng of *in vitro* transcribed renilla luciferase (rLuc) RNA was added to each aliquot as a spike-in control. Transcription reactions were performed with a mMessage mMachine T7 Transcription Kit according to manufacturer’s instructions and RNA was purified with acid phenol:cholorform extraction (Invitrogen). After adding the rLuc spike-in, 600 µL of guanidine HCl and 600 µL isopropanol were added to 400 µL of each fraction and incubated overnight at −20°C. Fractions were centrifuged at 10,000 g for 25 min to isolate RNA pellets. Samples were washed with 70% EtOH and resuspended in 400 µL of TE buffer. Cleanup was performed by precipitation with 40 µL of NaOAC and 2.5 volumes of 100% EtOH. Samples were centrifuged for 25 min at 10,000xg, pellets were washed with 70% EtOH, dried, and resuspended. Fractions corresponding to free RNA, 80S, disome/trisome, and dense polysomes were pooled and the RNA was then treated with RQ1-DNase (Promega) and reverse transcribed with Protoscript II Reverse Transcriptase (NEB), both according to manufacturer’s instructions. qPCR measurements with SYBR green were performed with the cDNA libraries and primers designed for each respective gene. The 18S rRNA primer set was adopted from Ref. [66]. Ct values for the rLuc spike-in were used to normalize variance in cDNA concentration arising due to sample cleanup and reverse transcription efficiency.

## S35 methionine and autoradiography

15 mL of TAP-tagged strains was grown in SC media lacking histidine (SC -His) to an OD_600_ of 0.4. Two cultures of *HSP30-TAP* and *PGK1-TAP* of equal OD were then mixed to make 30 mL. Cultures were pelleted, resuspended and grown in SC-His and 0.01x methionine for 30 min. To 15 mL of this combined culture, 0.2 mCi of [^35^S] methionine-cysteine (EXPRESS[^35^S] protein labelling mix; Perkin-Elmer) was added and incubated at 30°C for 30 min. To the remaining 15 mL, cells were pelleted and resuspended in SC-G, SC-His, 0.01x Met + 0.2 mCi [^35^S] and incubated at 30°C for 30 min. Labelled cells were pelleted and lysed in 400 µL RIPA buffer (50 mM Tris pH 8, 1% NP-40, 0.1% SDS, 0.5% Sodium Deoxycholate, 150 mM NaCl) with glass beads. Supernatants were isolated before being applied to immunoprecipitation with IgG-coupled beads. Dynabeads M270 Epoxy were coupled with IgG as described previously (https://commonfund.nih.gov/sites/default/files/Conjugation-of-Dynabeads.pdf).

Supernatants were incubated with Dynabeads for 30 min at RT, then washed 3 times with RIPA buffer. The Dynabeads were then resuspended in 25 μl of 1× loading buffer (50 mM Tris, pH 7.0, 2.5% sodium dodecyl sulphate (SDS), 0.02% bromophenol blue, 10% glycerol), and TAP-tagged proteins were eluted from the beads with moderate heat treatment at 65°C for 10 min. Loading buffer was transferred to a new tube, and 2-β-mercaptoethanol was added to a final concentration of 200 mM. Samples were boiled for 5 min, and 20 μl was loaded and resolved on 4–20% polyacrylamide gradient gels followed by autoradiography and quantitation with a PhosphorImager (Molecular Dynamics). Signal intensity was quantified using background subtraction and the ‘rectangles’ option in Quantity One software (Bio-Rad).

## Nanoluciferase reporter assays

Nluc assays were adapted from methods previously described [46]. Briefly, cells were grown in SC media and added to a 96-well plate. Promega Nano-Glo substrate was diluted 1:100 with PBS and added 1:10 to each well immediately prior to measurement. Luminescence was measured every 30 s with a Tecan Infinite 200 PRO plate reader. For glucose starvation, cells were sedimented by centrifugation, washed 2x with SC-G media and resuspended in SC G- media for 30 min of incubation at 30°C with rotating. 2% glucose was added with the substrate to monitor expression upon glucose readdition. For CHX-treated samples, 10 mg/mL CHX in deionized H_2_O was added to achieve a final concentration of 100 µg/mL. For LTM-treated samples, 3.5 mM LTM in DMSO was added to achieve a final concentration of 3.5 µM. To measure elongation rates during the diauxic shift, log phase cultures were inoculated in YPD media at 0.1 OD_600_ and incubated overnight for 24 hours. Assays were performed on the cultures at the indicated timepoints afterwards using the same methods described above.

## Elongation Measurements

Doxycycline was added to a final concentration of 10 mg/mL to induce transcription of the LacZ-Nluc and nLuc reporters in liquid culture. Luciferase expression was monitored as described in the preceding section. Data was linearized using Schleif plots to estimate the minimum reaction time required for complete translation [47]. The reaction time of the Nluc reporter was subtracted from the reaction time of LacZ-Nluc to calculate the time required for translation of the LacZ sequence alone. An RNA transcription speed of 2000 nt/min was used to calculate the estimated time required to transcribe the LacZ sequence [67]. Subtracting the transcription time from the LacZ reaction time provided the elongation rate for LacZ.

## Yeast gene length calculations

Median yeast gene length was calculated from information retrieved from SGD on 21 June 2021 (https://yeastmine.yeastgenome.org/yeastmine/bagDetails.do?scope=all&bagName=Verified_ORFs). The median length was calculated from the list of 5,195 genes categorized as verified ORFs.

## Supplementary Material

Supplemental Material
