# Supplementary material for: Differential translation elongation directs protein synthesis in response to acute glucose deprivation in yeast
Source: RNA Biol. 2022 May 1;19(1):636–49. doi: 10.1080/15476286.2022.2065784 (PMC9067459; doi:10.1080/15476286.2022.2065784)
Supplement: Supplemental Material [file KRNB_A_2065784_SM4023.docx]

# Supplemental Material:


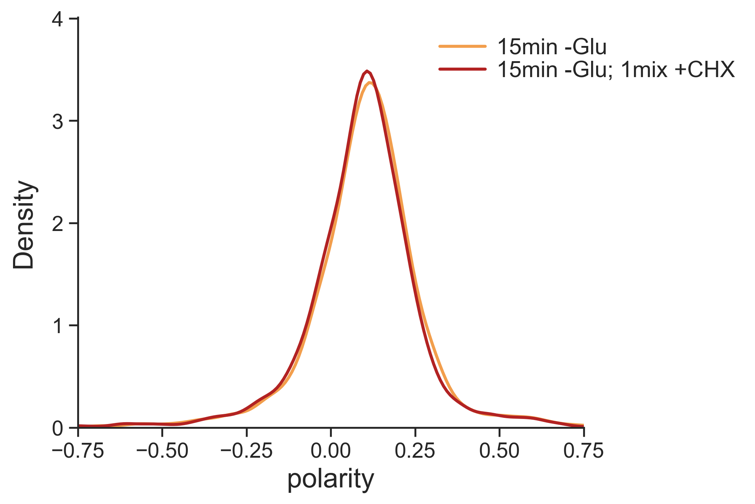

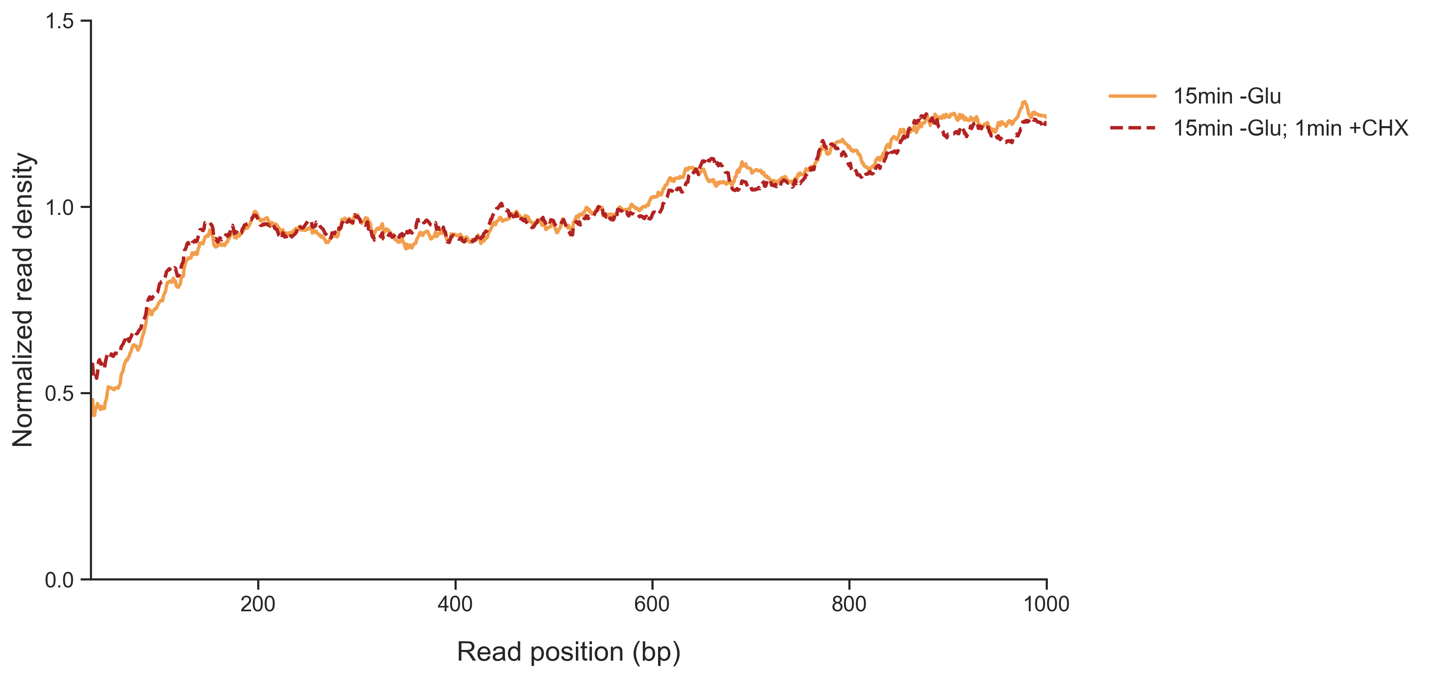


**Figure S1**: *Cycloheximide pretreatment does not alter ribosome distribution or polarity after fifteen minutes of glucose starvation.*

Normalized read density plots generated from the aggregate number of reads per nucleotide position across all genes >1000nt with > 25 reads per gene per library (top). Densities of polarity score distributions from the indicated samples (bottom). Polarity scores were calculated and included in the distributions for all genes that had > 25 reads per library. Libraries were prepared without CHX pretreatment.

**A**

**
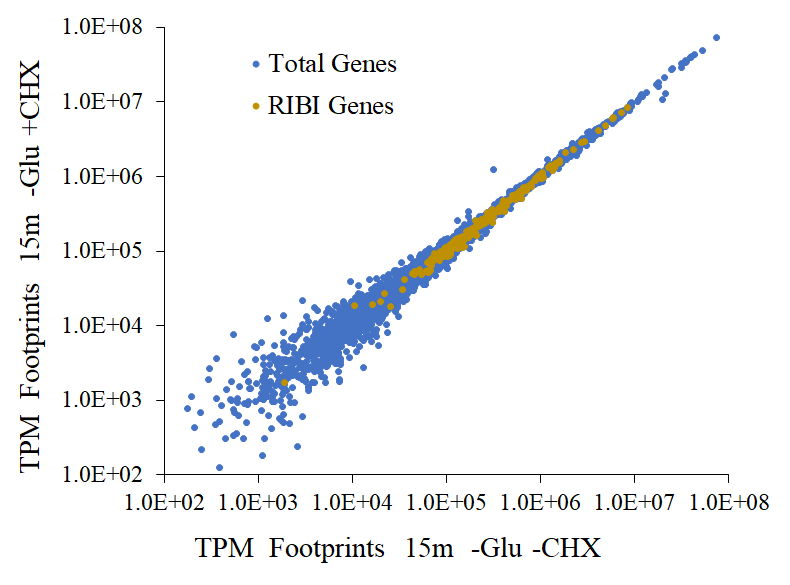
**

**B**


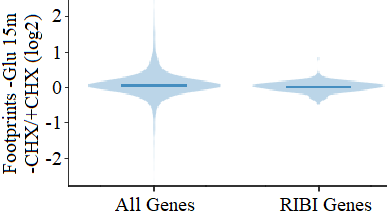


**Figure S2**: *Effect* of c*ycloheximide pretreatment on ribosome reads after fifteen minutes of glucose starvation.*

**A.** Scatterplot of footprints for each gene with > 100 TPM in CHX pretreatment or no CHX pretreatment samples. **B**. Violin plot of normalized footprint ratios for All genes and RIBI genes. No significant difference was seen between these two groups.


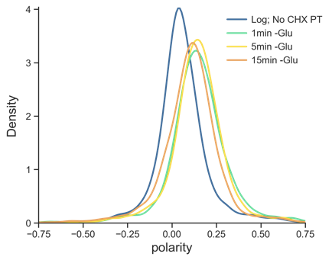


**Figure S3**: *Polarity score analysis over a time course of acute glucose starvation.*

Densities of polarity score distributions from the indicated log phase and glucose starvation time course samples. Polarity scores were calculated and included in the distributions for all genes that had > 25 reads per library. Libraries were prepared without CHX pretreatment.


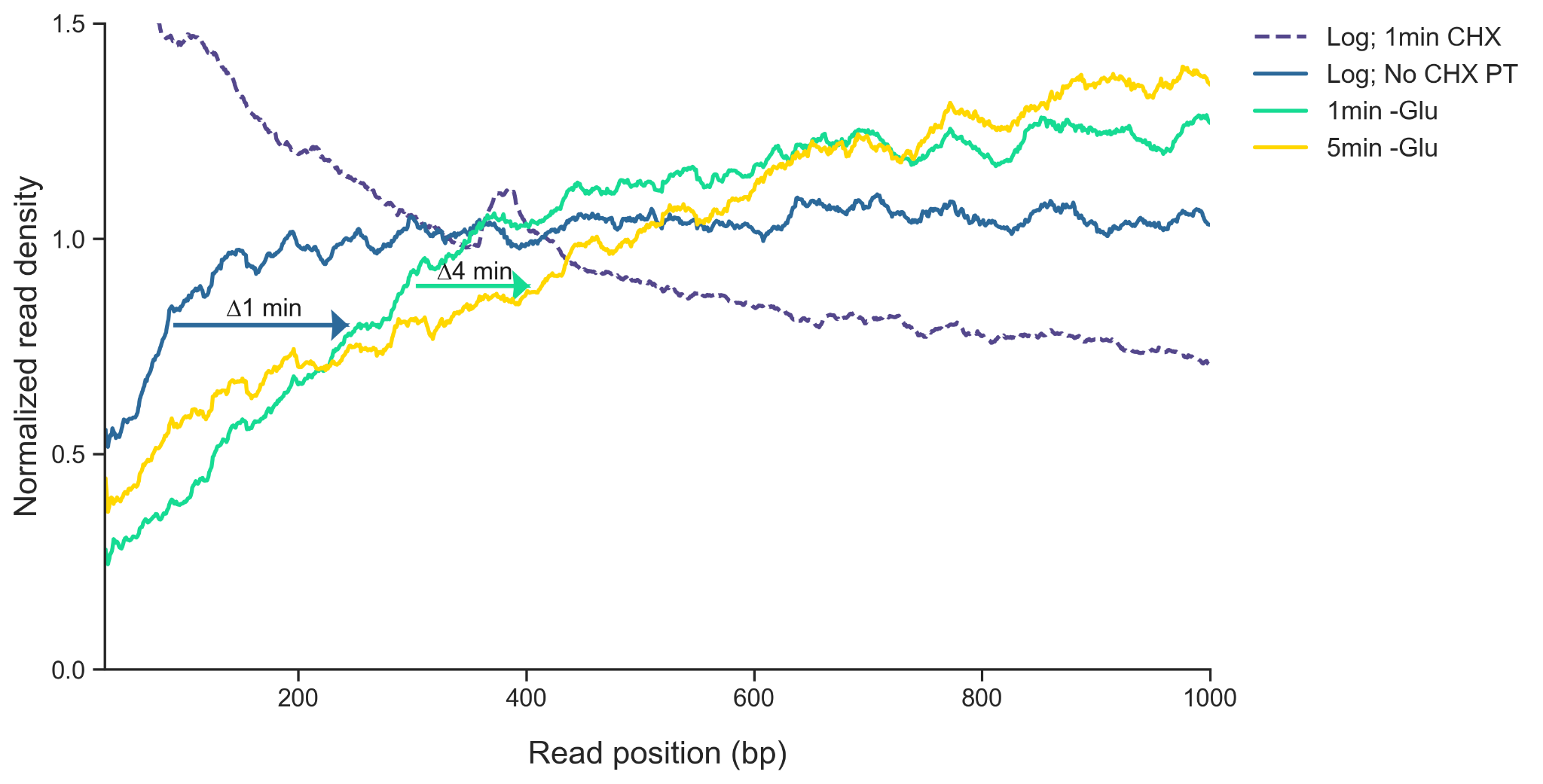


**Figure S4**: *Read density near the start codon is impacted by CHX-pretreatment and its decrease slows progressively over a time course of acute glucose starvation*.

Normalized read density plots generated from the aggregate number of reads per nucleotide position across all genes >1000nt with > 25 reads per gene per library. Arrows indicate the amount of time elapsed between sample collection. For the Log; No CHX PT sample to 1min -Glu sample there is a ∆1min while the 1min -Glu to 5min -Glu samples have ∆4min. Arrows were drawn at the read-density position on the y-axis that is halfway between the minimum and maximum read density score from the Log; No CHX PT and 1min -Glu samples, respectively. Arrows are intended to be visual aids to show how the vacancy of ribosome read density is greater between the Log: No CHX PT to 1min -Glu sample while less time elapsed between collection (1minute versus 4 minutes).


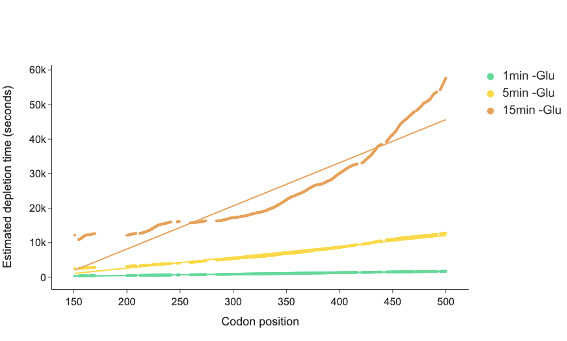


**Figure S5**: *Ribosome depletion time calculations show a progressive slowing of ribosome transit in response to acute glucose starvation.*

Ribosome profiling reads were used to estimate the depletion time required for ribosomes to move along genes as a function of codon position based on the relative movement of read density between samples collected at different time points. Methods to calculate depletion time were adopted from (Sharma et al., 2019). For each time point, the relative ribosome density at each codon position is calculated by comparing ribosome density at glucose starvation to log phase conditions. This value is then used to estimate the time needed for ribosome depletion. The straight lines show the fitted data using a linear model. The coefficients of determination (R^2) of the fitted lines for 1 minutes, 5 minutes, and 15 minutes are 0.996, 0.970, and 0.862, respectively.


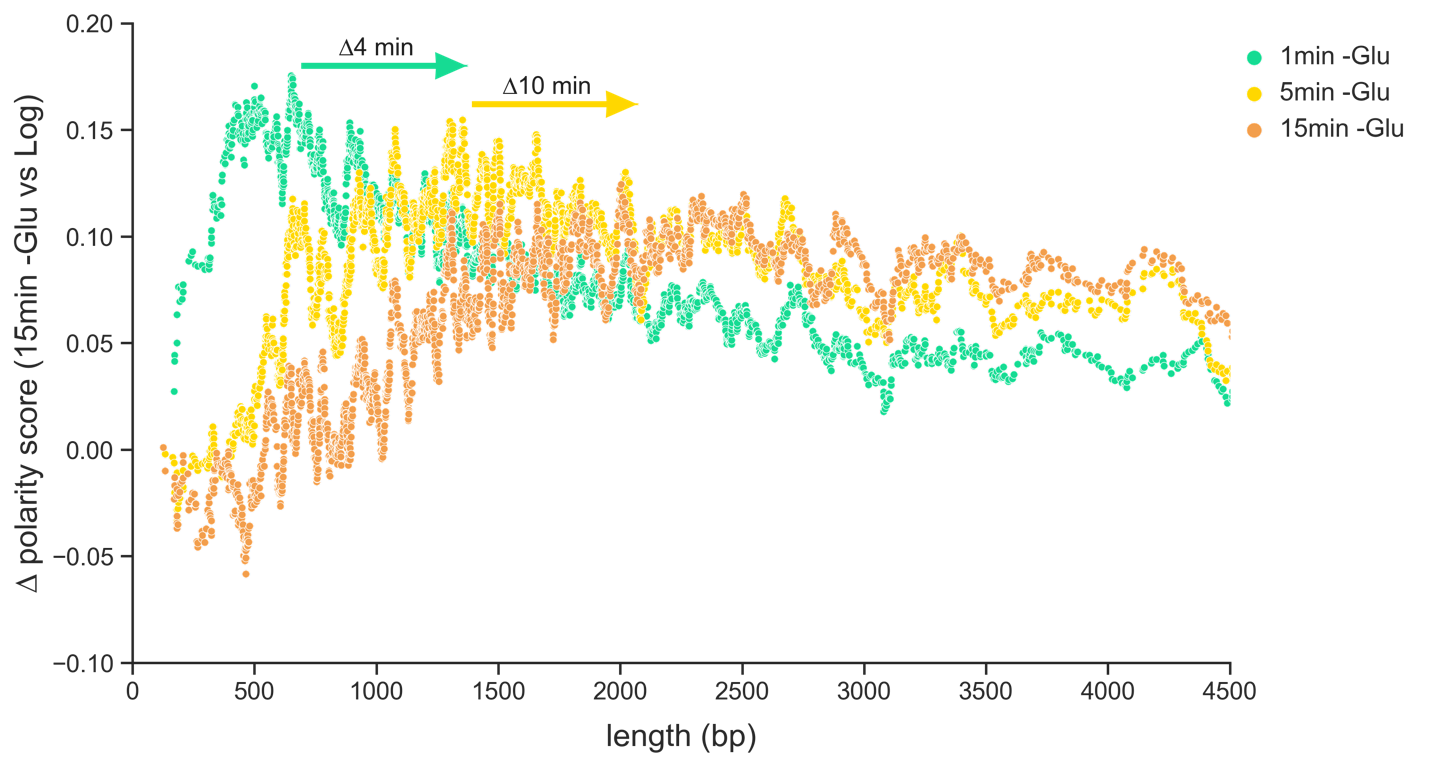


**Figure S6**: *Polarity score changes plotted against gene length after acute glucose starvation show a slowing of ribosome movement over time.*

For the top 2,500 expressed genes as assessed by RPF read TPM in log phase, genes were sorted by length and their change in polarity score (∆ polarity score) was calculated for each indicated time course sample by subtracting the polarity score for the same gene in log phase (without CHX pretreatment). The rolling average with a 30nt window of the ∆ polarity score was plotted against gene length. Arrows were drawn at the position on the y-axis where the change in polarity score is greatest for the indicated sample. As in Figure S2, arrows are intended to be visual aids to indicate how the greatest positive change in score is similar between the Log: No CHX PT to 1min -Glu sample even though less time elapsed between collection (4 minutes versus 10 minutes).

**
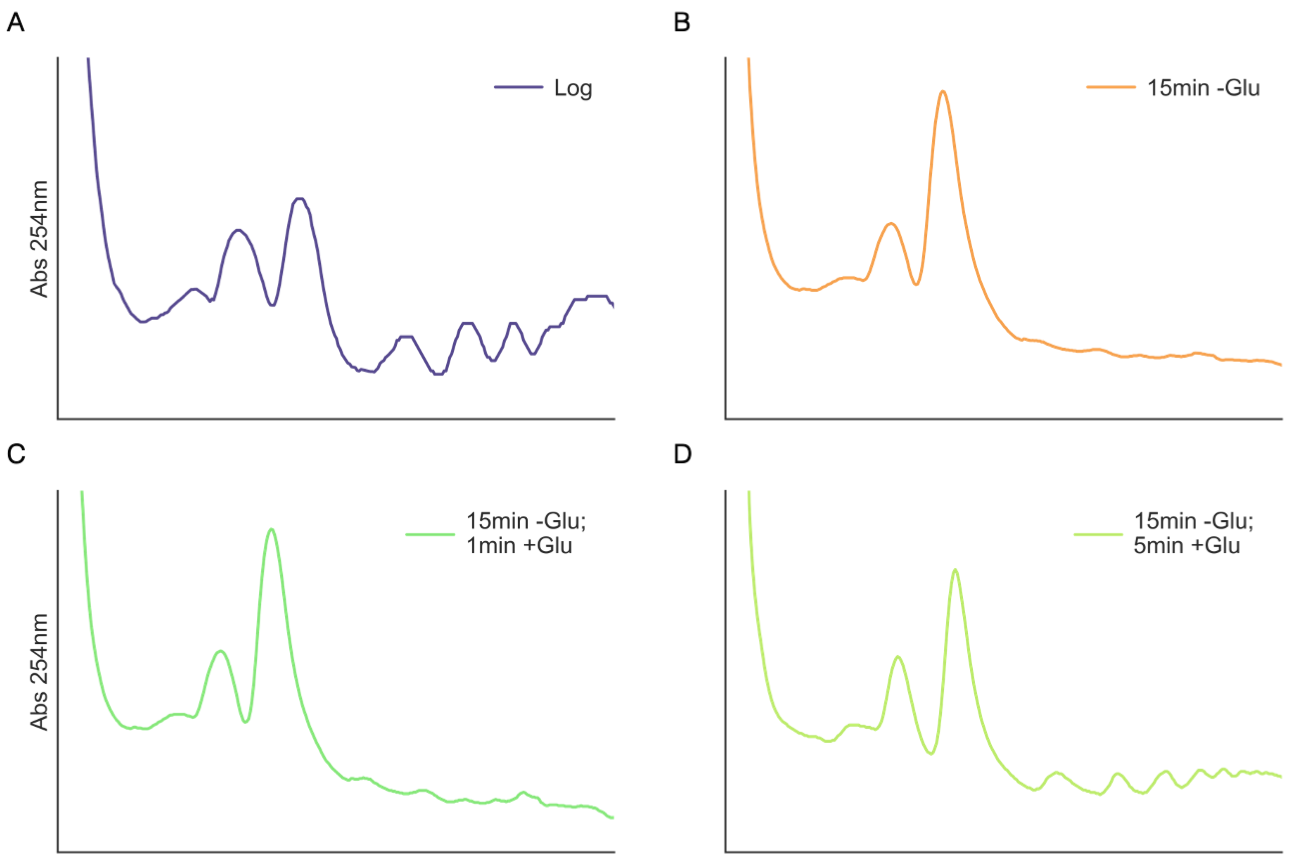
**

**Figure S7**: *Polysome traces from log phase, glucose starved, and glucose readdition samples.*

﻿**A**: Sedimentation profile of cells grown to log phase. **B**: Sedimentation profile of log phase cells that underwent 15 minutes of glucose starvation. **C**: Sedimentation profile of log phase cells that underwent 15 minutes of glucose starvation and then were supplemented with glucose for one minute. **D:** Sedimentation profile of log phase cells that underwent 15 minutes of glucose starvation and then were supplemented with glucose for 5 minutes.

**
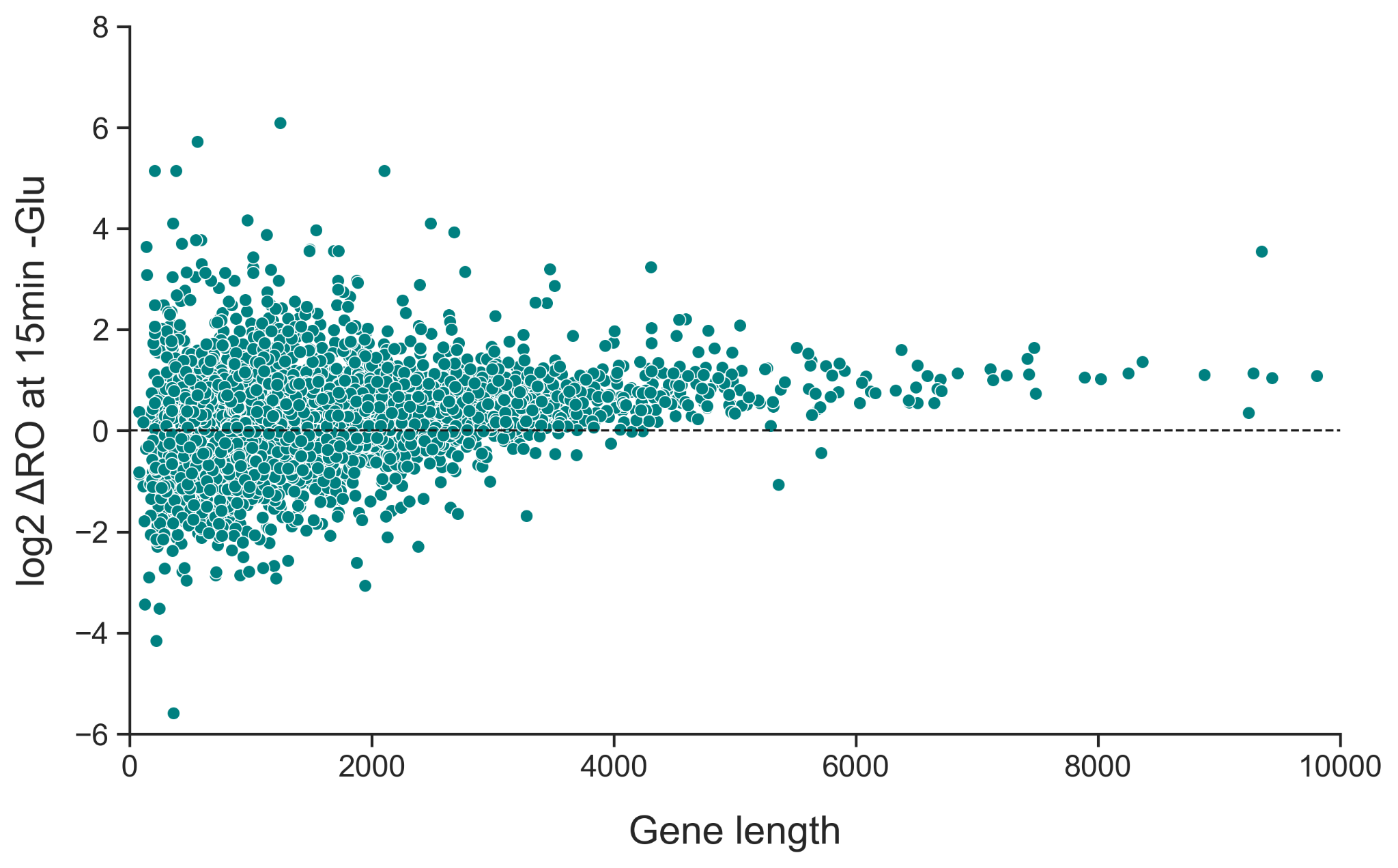
**

**Figure S8**: *Ribosome occupancy change after 15 minutes glucose starvation against gene length shows longer genes have higher relative occupancy during stress.*

RO was calculated per gene as RPF reads divided by mRNA reads for the same gene. Log2 values of the difference in RO score per gene between 15 minutes of glucose starvation and log phase (y-axis) were plotted against gene length in nucleotides (x-axis).

**Table S1: Yeast strains used in this study**

| **Strain** | **Genetic Background** | **Reference source** |
| --- | --- | --- |
| BY4741 | MATa his3Δ1 leu2Δ0 met15Δ0  ura3Δ0 | Euroscarf |
| EY0690 | MATa trp1-1 leu2-3 ura3-1 his3-11 can1-100 | W303 |
| P_TetO7_-Nluc only reporter | BY4741, P_ERV14_-rtTA::URA3, P_TetO7_-NlucPEST-MS2(v4)::HIS3 | This study |
| P_TetO7_-LacZ-Nluc reporter | BY4741, P_ERV14_-rtTA::URA3, P_TetO7_-LacZ-NlucPEST-MS2(v4)::HIS3 | This study |
| Pgk1 TAP tag | S288C: (ATCC 201388: MATa his3Δ1 leu2Δ0 met15Δ0 ura3Δ0) | Yeast-TAP Tagged ORF library collection (Horizon Discovery) |
| Hsp30 TAP tag | S288C: (ATCC 201388: MATa his3Δ1 leu2Δ0 met15Δ0 ura3Δ0) | Yeast-TAP Tagged ORF library collection (Horizon Discovery) |
| ZY185 | EY0690, HIS3 OsTIR1, tTA, TetR’-SSN6, Dhh1-3xmini-AID-5xFlag-KanMX | This study |
| Fas1-E2A-NlucPEST | EY0690, Fas1::E2A-NlucPEST::HIS3 | This study |
| Ura2-E2A-NlucPEST | EY0690, Ura2::E2A-NlucPEST::HIS3 | This study |

**Table S2: qPCR primers used in this study**

| **qPCR Gene and Primer Set** | **Forward Primer** | **Reverse Primer** |
| --- | --- | --- |
| 18S rRNA (ZO995/996) | AATCATCAAAGAGTCCGAAGACATTG | CCTTTACTACATGGTATAACTGTGG |
| Acc1 (ZO1014/1015) | TTTCTGCCATTTTCTCTACTCC | TGTTCAGTTCTTTCCTTGACC |
| Act (ZO83/84) | CTGCCGGTATTGACCAAACT | CGGTGATTTCCTTTTGCATT |
| Fas1 (ZO787/788) | CGCTGCATCATTCTCTCAAG | TTGACGATTTCAACCAACCA |
| Hsp30 (OS262/263) | TTGGACTGGTGTTCAAGCTG | CAGGACAAGAACCAGGCAAT |
| Hsp104 (OS803/804) | CGACGCTGCTAACATCTTGA | CACTTGGTTCAGCGACTTCA |
| Pab1 (ZO95/96) | TCTCTGTGTTTGGTGACATCTT | TTGGCAGCACCTTCTTCTT |
| Pgk1 (OS773/774) | GGACAAGCGTGTCTTCATCA | CGTTTCTTTCACCGTTTGGT |
| RPS8A (ZO952/953) | TCAACCAGCCAACACCAAG | CAGAAGCCCAAGAAAAGTTACC |
| Ura2 (ZO1018/1019) | ATTCCCCGCTTACACGAAC | AACACCAGAACCCAAGACC |
